# Isolation, Identification, and Pathogenicity of Entomopathogenic Fungal Strains and Their Secondary Metabolites Against *Spodoptera frugiperda* (Lepidoptera: Noctuidae)

**DOI:** 10.1007/s13744-026-01360-3

**Published:** 2026-02-18

**Authors:** Fatmaalzahraa A. Senousy, El Arnaouty S.A, Mona N. Kortam, Ismail M. Ismail, Mona Awad, Marwa A. Zayton

**Affiliations:** 1 Dept of Economic Entomology and Pesticides, Faculty of Agriculture, Cairo Univ, Giza, Egypt; 2 Central Lab of Organic Agriculture, Agricultural Research Center, Giza, Egypt; 3 Dept of Microbial Molecular Biology, Agricultural Genetic Engineering Research Institute (AGERI), Agriculture Research Centre, Giza, Egypt; 4 Dept of Plant Pathology, Faculty of Agriculture, Cairo Univ, Giza, Egypt

**Keywords:** Fall armyworm, fungal secondary metabolites, microbial control, oxidative stress enzymes, virulence

## Abstract

The fall aِrmyworm, *Spodoptera frugiperda* (J.E. Smith), causes significant yield losses due to its high fecundity, broad host range, defoliation capability, and voracious larval feeding. Biological control using microorganisms has gained attention as a sustainable alternative to chemical pest management. Entomopathogenic fungi employ distinct modes of action and produce secondary metabolites with insecticidal properties. This study aimed to isolate and identify indigenous entomopathogenic fungal species and evaluate their pathogenicity against *S. frugiperda*. Three isolates belonging to the division Ascomycota were recovered from soil samples and identified as *Botryotrichum domesticum* isolate MP3H-5, *Albifimbria verrucaria* isolate E16, and *Purpureocillium lilacinum* isolate WARSO2 6 8. When applied at a concentration of 1 × 10^9^ conidia mL⁻^1^, the fungal isolates induced significant cumulative mortality (60 to 84.44%) in *S. frugiperda* larvae at 14 days post-treatment. *Albifimbria verrucaria* was the most virulent, exhibiting the lowest LC_50_ (2 × 10^7^ conidia mL⁻^1^) and LT_50_ (0.46 days). At a lower concentration of 1 × 10^7^ conidia mL⁻^1^, the *P. lilacinum* isolate caused the highest pupal mortality (80%) at 7 days post-treatment. Crude fungal metabolites also demonstrated larvicidal activity, resulting in cumulative mortality rates of up to 50%. Furthermore, larvae treated with the entomopathogenic fungi showed elevated catalase activity. The high toxicity observed is likely associated with fungal secondary metabolites, which were characterized using GC-MS analysis. Under laboratory conditions, the tested entomopathogenic fungal isolates demonstrated strong potential as microbial agents for the control of *S. frugiperda*. However, further field-based validation is necessary to confirm their efficacy.

**Supplementary Information:**

The online version contains supplementary material available at 10.1007/s13744-026-01360-3.

## Introduction

Maize *Zea mays* (Linnaeus), a member of the kingdom Plantae and family Poaceae, is a major cereal crop globally and the second most important strategic crop in Egypt (Gao et al. 2020). However, maize yield is severely affected by various biotic stresses, particularly phytophagous insect pests such as root feeders, stem borers, phloem feeders, and leaf feeders (Tefera et al. 2019; Badji et al. 2020). Among these, the fall armyworm, *Spodoptera frugiperda* (J.E. Smith) (Lepidoptera: Noctuidae), has emerged as a serious threat to food security in Africa due to its polyphagous nature and voracious feeding behavior, causing substantial losses in maize production (Badji et al. 2020; Gómez-Valderrama et al. 2022). *Spodoptera frugiperda* was first introduced into Africa through West and Central African countries (Goergen et al. 2016) and has since spread rapidly to 109 countries (Desika et al. 2024), with reported maize yield losses ranging from 20 to 50% (Day et al. 2017; Aruna Balla et al. 2019). In Egypt, *S. frugiperda* was officially reported on May 31, 2019, in maize fields in the Aswan Governorate and subsequently expanded into northern regions (Mohamed et al. 2022).

Effective management of *S. frugiperda* is challenging due to its wide host range, high reproductive capacity, aggressive behavior, intense feeding activity, and rapid migration (Mooventhan et al. 2019; Jaba et al. 2020). Control strategies have primarily relied on synthetic insecticides (Trdan et al. 2020). Commonly used chemical insecticides include organophosphates, pyrethroids, and neonicotinoids, which provide rapid action. However, excessive use of these chemicals has resulted in environmental pollution, the development of insecticide resistance, and harmful effects on soil and non-target organisms (Desika et al. 2024). As a result, biological control using natural enemies such as predators, parasitoids, and entomopathogens has gained attention as a promising alternative (Kenis 2023). Entomopathogens include bacteria, viruses, fungi, protists, and nematodes capable of infecting and ultimately killing their insect hosts (Karthi et al. 2024).

Among these, entomopathogenic fungi (EPF) are unique microbial agents that infect insects through direct penetration of the cuticle, offering a significant advantage in integrated pest management (IPM) programs (Qin et al. 2023). The EPF spores adhere to the insect cuticle, germinate, and penetrate the integument. Once inside the hemocoel, they proliferate, invade internal tissues, deplete nutrients, and secrete toxic secondary metabolites that contribute to host mortality (Rajula et al. 2021). Members of the genus *Botryotrichum*, for instance, are known to produce lipolytic enzymes, particularly lipases which facilitate fungal penetration by degrading lipids in the insect cuticle, thereby enhancing infectivity (Moharram et al. 2021). Likewise, the fungus *Albifimbria verrucaria* produces chitinases that degrade chitin, a major component of the insect exoskeleton and fungal cell walls. These enzymes exhibit strong larvicidal and anti-feeding activity against insects and antifungal activity against plant pathogens, positioning *A. verrucaria* as a promising biocontrol agent for both insect pests and fungal diseases (Vidhate et al. 2019).

In response to pathogen invasion, the insect immune system generates elevated levels of reactive oxygen species (ROS) (Kaur et al. 2021). However, excess ROS induces oxidative stress, disrupting the balance between antioxidants and pro-oxidants in insect cells and resulting in immunotoxicity and oxidative damage (Kaur et al. 2020; Krishnan and Sehnal 2006). To mitigate oxidative stress, insects regulate ROS levels through the activity of antioxidant enzymes. Among these, catalase (CAT) plays a central role by converting hydrogen peroxide (H_2_O_2_) into water (H_2_O) and molecular oxygen (O_2_), thereby protecting cells from oxidative damage under high steady-state ROS conditions (Ghanem et al. 2020).

The present study aimed to (1) isolate and identify indigenous EPF from soil samples, (2) evaluate their virulence against *S. frugiperda* larvae and pupae, (3) assess the toxicity of their crude secondary metabolites against *S. frugiperda* larvae, and (4) investigate the enzymatic response of *S. frugiperda* following infection with the isolated EPF strains.

## Materials and methods

### Isolation of entomopathogenic fungi from soil

Soil samples (approximately 200 g each) were collected from two locations in Giza Governorate, Egypt: (30°1′5″N, 31°12′32″E) and (30°02′N, 31°13′E). Samples were randomly collected in clean, sterile plastic cups after removing the top 5 cm of the soil surface and were stored at 4 °C until further use (Tuininga et al. 2014). The *Galleria* Bait Method (Zimmermann 1986) was used for the isolation of EPF. Ten larvae of *Galleria mellonella* L. (Lepidoptera: Pyralidae) were placed in a plastic container with 50 g of each soil sample, along with moistened filter paper discs, and incubated at 25 ± 2°C. Larval mortality and fungal growth were monitored daily (Herlinda and Mulyati 2008). Upon observing fungal growth, the infected larvae were treated with 1% sodium hypochlorite solution for 2 min, rinsed three times with distilled water, air-dried, and then were aseptically transferred onto sterilized Sabouraud Dextrose Agar (SDA) plates (9 cm diameter) and incubated at 25 ±  1 °C for 14 days (Goettel and Inglis 1997; Russo et al. 2021; Beemrote et al. 2024). Fungal isolates were purified using the hyphal tip technique (Herlinda et al. 2020).

### Morphological characterization of EPF candidates

The isolated fungi were identified at the Department of Fungal Classification and Identification, Plant Pathology Research Institute, Agricultural Research Center, Giza, Egypt. Identification was based on cultural and morphological characteristics, including colony growth patterns and morphology. For microscopic examination, slides were prepared from each isolate and examined under a Leica light microscope. Conidial morphology and structure were characterized according to the guidelines of Mongkolsamrit et al. (2020).

### Molecular identification and phylogenetic analysis

Molecular identification of the three most promising isolates was performed through PCR amplification of the internal transcribed spacer (ITS) region. PCR reactions were carried out in a 50-µL volume containing 25-µL Master Mix, 2 µL of each primer (forward and reverse, 10 pmol), 3-µL template DNA (10 ng), and 15- µL distilled water. Amplification followed the protocol of White et al. (1990) using primers ITS1 F (5′-GCATCGATGAAGAACGCAGC-3′) and ITS4 R (5′-TCC TCC GCT TAT TGA TAT GC-3′). PCR amplification was conducted as described by Madbouly et al. (2020). Products were separated on a 1.5% agarose gel containing ethidium bromide (0.5 µg/mL) in 1× TBE buffer and electrophoresed at 95 V (Fig. S1). The PCR products were purified using the High Pure PCR Clean-Up & Gel Extraction Kit (GeneDireX), according to the manufacturer’s instructions. DNA sequencing was performed, and the resulting sequences were analyzed using the NCBI Basic Local Alignment Search Tool (BLAST) (http://www.ncbi.nlm.nih.gov/BLAST). Sequences were aligned and compared to reference rDNA sequences available in GenBank. Phylogenetic analysis was conducted using MEGA11 software. Evolutionary relationships were inferred using the Neighbor-Joining method with 1000 bootstrap replications (Ghoneem et al. 2023).

### Pathogenicity bioassays

#### Insect culture

*Spodoptera frugiperda* larvae were obtained from the Department of Entomology, Faculty of Agriculture, Cairo University, Egypt. The colony was maintained under control conditions (26°C ±  2 °C, relative humidity of 65 ± 5%, and a 16-h light:8-h dark photoperiod). Neonates were fed fresh castor oil leaves (*Ricinus communis* Linnaeus; kingdom Plantae, family Euphorbiaceae); from the third instar onward, larvae were reared individually in small cups to prevent cannibalism, and a mass-rearing colony was maintained as described by Moustafa et al. (2025). Pupae were monitored daily until adult emergence. After emergence, adult moths (seven males and five females) were transferred to a larger jar lined with cotton wool soaked in 10% sugar solution. Eggs were collected and transferred to clean jars for hatching. Second-instar larvae were used for all experimental treatments.

#### Preparation of conidial suspensions

Conidia were harvested by adding 5 mL of sterile 0.03% Tween 80 to each fungal culture and dislodging the spores using a sterile spreader. The resulting suspension was filtered through four layers of sterile cheesecloth. Conidial concentrations were adjusted to 1 × 10^6^, 1 × 10^7^, 1 × 10^8^, and 1 × 10^9^ conidia mL⁻^1^ using a Neubauer hemocytometer (Brand GmbH, Wertheim, Germany).

#### Pathogenicity of tested isolates against the larval stage of *S. frugiperda*

Bioassays were conducted by dipping second-instar *S. frugiperda* larvae in each concentration of fungal conidial suspension (1 × 10^6^, 1 × 10^7^, 1 × 10^8^, and 1 × 10^9^ conidia mL⁻^1^) for 10 s. For each concentration, 50 larvae (10 larvae × 5 replicates) were used. Control larvae were dipped in distilled water containing 0.03% Tween 80 under the same conditions. Each larva was individually placed in a sterile plastic cup and provided with fresh castor leaves daily. Larvae were maintained under laboratory conditions, and mortality was recorded at 3, 5, 7, 9, 12, and 14 days post-treatment. Larvae were considered dead if they did not respond to gentle probing with forceps (Ramanujam et al. 2020).

To confirm fungal-induced mortality, a mycosis test was performed. Cadavers were surface sterilized in 70% ethanol, followed by three rinses with sterile distilled water. Each disinfected larva was placed in a Petri dish lined with sterile, moistened filter paper and incubated at 25 ±  2 °C in the dark. Fungal infection was confirmed by observing hyphal and spore emergence from the cadavers (Louw et al. 2025).

#### Pathogenicity of the tested isolates against the pupal stage of *S. frugiperda*

One-day-old pupae of *S. frugiperda* were immersed for 10 s in conidial suspensions of the tested isolates at concentrations of 1 × 10^6^ and 1 × 10^7^ conidia mL⁻^1^. Control pupae were dipped in distilled water containing 0.03% Tween 80. For each concentration, 15 pupae (five pupae per replicate) were individually maintained in clean plastic cups. Pupal mortality was monitored and recorded at 3, 5, and 7 days post-treatment. To verify that mortality was caused by fungal infection, a mycosis test was conducted following the method of Liu et al. (2022a, b), which involved assessing pupal response to touch and observing characteristic symptoms such as failure to melanize or emerge.

### Metabolite profiling of entomopathogenic fungal isolates

#### Production and extraction of secondary metabolites

Fungal cultures (200 mL) were prepared in sterile 500-mL Erlenmeyer flasks containing Potato Dextrose Broth (PDB). Each flask was inoculated with a 7-day-old fungal agar disc, placed to float on the medium surface. Triplicate cultures were prepared for each isolation and incubated at 25 °C for 20 days. Following incubation, the culture filtrates were extracted three times with methylene chloride, followed by three extractions with ethyl acetate using a separating funnel. The ethyl acetate layer was filtered through anhydrous Na_2_SO_4_ and evaporated to dryness to obtain crude metabolite residues.

#### GC-MS analysis

The chemical composition of the extracts was analyzed using a Trace GC-TSQ mass spectrometer (Thermo Scientific, Austin, TX, USA) equipped with a TG–5MS capillary column (30 m × 0.25 mm × 0.25 µm film thickness). The column oven temperature was initially set at  50 °C, then increased by  5 °C per minute to 250 °C (held for 2 min), followed by a ramp of 30 °C per min to a final temperature of 300 °C (held for another 2 min). Helium served as the carrier gas at a constant flow rate of 1 mL/min. The injector and MS transfer line were maintained at  270 °C and  260 °C, respectively. One microliter of each diluted sample was injected automatically into split mode using the Autosampler AS1300, following a 4-min solvent delay. Massmass spectra were acquired in full-scan mode (m/z 50–650) with an electron ionization voltage of 70 eV. The ion source temperature was maintained at  200 °C, as compound identification was achieved by comparing the obtained spectra with the NIST 14 and WILEY 09 spectral libraries.

### Bio-efficacy of secondary metabolites of entomopathogenic fungi isolates against *S. frugiperda* Larvae

Crude fungal metabolites from each EPF isolate were evaluated against newly ecdysed second-instar larvae of *S. frugiperda* using the leaf-dip technique described by Yue et al. (2022). Castor leaf discs (5 cm diameter) were immersed in each crude extract for 20 s, air-dried, and placed in individual plastic cups. Each treatment consisted of 50 larvae (10 larvae per replicate). Leaf discs treated with distilled water served as the control. Larvae were maintained individually under the same controlled conditions described earlier and were provided with fresh castor leaves daily. Mortality was recorded every 3 days for a total of 14 days.

### Biochemical analysis

#### Oxidative stress enzyme assays

##### Sample preparation

Second-instar larvae of *S. frugiperda* were treated with the highest conidial concentration of 1 × 10^9^ conidia mL⁻^1^ and metabolites of the three isolates using the above-described method. At 3 and 5 days post-treatment, 100 mg of fresh larval body weight was collected into sterile 1.5-mL Eppendorf tubes and immediately stored at −20°C until use. Six replicates were prepared for each treatment. The samples were homogenized in potassium phosphate buffer (50 m, pH 7.0) at a ratio of 30 μL of buffer per 1 mg tissue. Homogenates were centrifuged at 7000×g for 15 min at 4 °C, and the supernatants were collected for enzymatic assays.

##### Enzyme measurement

Catalase (CAT) activity was measured by monitoring the rate of H_2_O_2_ decomposition using a commercial assay kit (Bio-Diagnostic Company, Giza, Egypt). The amount of H_2_O_2_ consumed decomposed H_2_O_2_ was measured spectrophotometrically at 510 nm according to the method of Aebi (1984). Total protein concentration was determined using the Biuret method and a Protein Biuret Kit (Bio-Diagnostic Company, Giza, Egypt).

### Data analysis

Larval mortality data were corrected using Abbott’s formula. All statistical analyses were performed using SPSS software (version 22). The data were assessed for normality using Shapiro–Wilk and Kolmogorov–Smirnov tests. Percentage data were normalized using arcsine square root transformation. Results are presented as mean ± standard deviation. One-way ANOVA was conducted to compare treatment effects across all groups (including controls), with at least three replicates per group. Tukey’s post hoc test was used for pairwise comparisons, and differences were considered statistically significant at *P* < 0.05. Data visualization was performed using R Studio (version 2025.05.0+496), where applicable (Maronna et al. 2019). Lethal time (LT_50_) and lethal concentration (LC_50_) values were calculated via probit analysis using LDP Line software, as described by Finney (1971).

## Results

### Morphological characterization

**Fig. 1:**
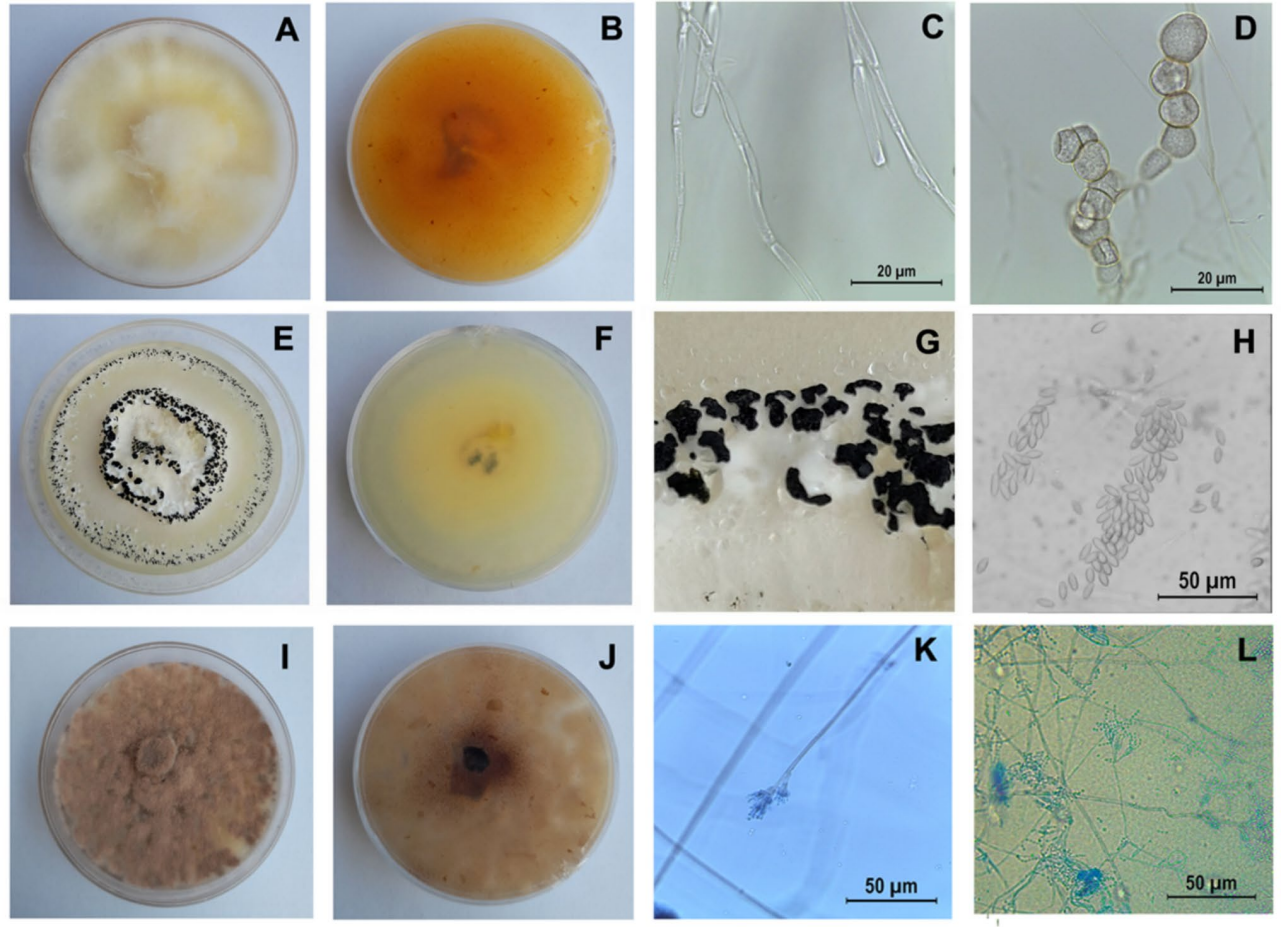
Morphological characteristics of the three tested fungi cultured on potato dextrose agar (PDA) at 25 °C for 7 days. **A**–**D** *Botryotrichum domesticum*: **A** colony morphology on the obverse; **B** colony reverse pigmentation; **C** septate, smooth-walled hyphae under light microscopy; **D** one-celled, globose to sub-globose conidia arranged in chains. Scale bars: 20 μm. **E**–**H**) *Albifimbria verrucaria*: **E** colony morphology showing dark spore masses surrounded by white mycelia; **F** colony reverse pigmentation; **G** close-up of the colony surface showing clustered black sporulation zones encircled by floccose white margins; **H** light micrograph of free, elongated, fusiform conidia. Scale bar: 50 μm. **I**–**L ***Purpureocillium lilacinum*: **I** colony morphology displaying woolly to floccose texture with violet to reddish-gray pigmentation; **J** colony reverse pigmentation; **K** conidiophore bearing whorled phialides under light microscopy; **L** conidia arranged in divergent chains. Scale bars: 50 μm

Three indigenous fungal strains were successfully isolated from soil samples collected in Giza Governorate, Middle Egypt. The first isolate was identified as *Botryotrichum domesticum* Li and Schultes (Eukaryota; Fungi; Dikarya; Ascomycota; Sordariomycetes; Sordariales; Chaetomiaceae). Colonies were smooth, circular, and planar with aerial mycelium, displaying a yellow to light beige coloration on PDA. Hyphae were septate, colorless, smooth, thin-walled, and aerial. Conidia were solitary, initially hyaline, one-celled, globose to sub-globose, and arranged in chains of coupled spores. Conidial surfaces ranged from smooth to slightly roughened (Fig. 1).

The second isolate was *Albifimbria verrucaria* (Albertini and Schweinitz), formerly *Myrothecium verrucaria* (Eukaryota; Fungi; Dikarya; Ascomycota; Sordariomycetes; Hypocreales; Stachybotryaceae). *Albifimbria verrucaria* is a saprophytic fungus with global distribution, inhabiting soil and decomposing plant debris. Colonies had white mycelium with a buff reverse. Hyphae were hyaline, smooth, thin-walled, and rarely branched. Spore masses ranged in color from very dark olive to nearly black, clustered in moist, convex structures bordered by a white, floccose margin. Spores were broadly fusiform, unicellular, hyaline to pale olivaceous, and appeared black in mass (Fig. 1).

The third isolate was *Purpureocillium lilacinum* Thom, formerly *Paecilomyces lilacinus* (Eukaryota; Fungi; Dikarya; Ascomycota; Sordariomycetes; Hypocreales; Ophiocordycipitaceae). This species, recognized for its potential as a biocontrol agent, formed colonies with hues ranging from vinaceous to violet or mauve, often turning reddish gray depending on the substrate. Colonies showed moderately rapid growth and had a woolly to floccosetexture. Conidiophores bore branches with densely clustered phialides. Conidia were ellipsoidal to fusiform, smooth- to slightly rough-walled, and hyaline (Fig. 1).

### Molecular identification

PCR amplification using ITS1/ITS4 primers produced fragments of approximately 650 bp, corresponding to the ITS1, 5.8S, and ITS2 regions of rDNA from the three selected fungal isolates (Fig. S1). Alignment analysis of the 650 bp sequences was conducted against the five most homologous sequences in the NCBI database. The sequences of the three isolates (designated FAMS 1, FAMS 2, and FAMS 3) were submitted to GenBank and assigned accession numbers: PP348706.1, PP348707.1, and PP348781.1, respectively.

Fungal isolate 1 (FAMS 1) shared 97% similarity with *B. domesticum* isolate MP3H-5 (GenBank accession MZ827841.1). Isolate 2 (FAMS 2) showed 100% identity with *A. verrucaria* isolate E16 (GenBank accession JQ356542.1). Isolate 3 (FAMS 3) displayed 96% similarity with *P. lilacinum* isolate WARSO2 6 8 (GenBank accession MN451854.1).

**Fig. 2:**
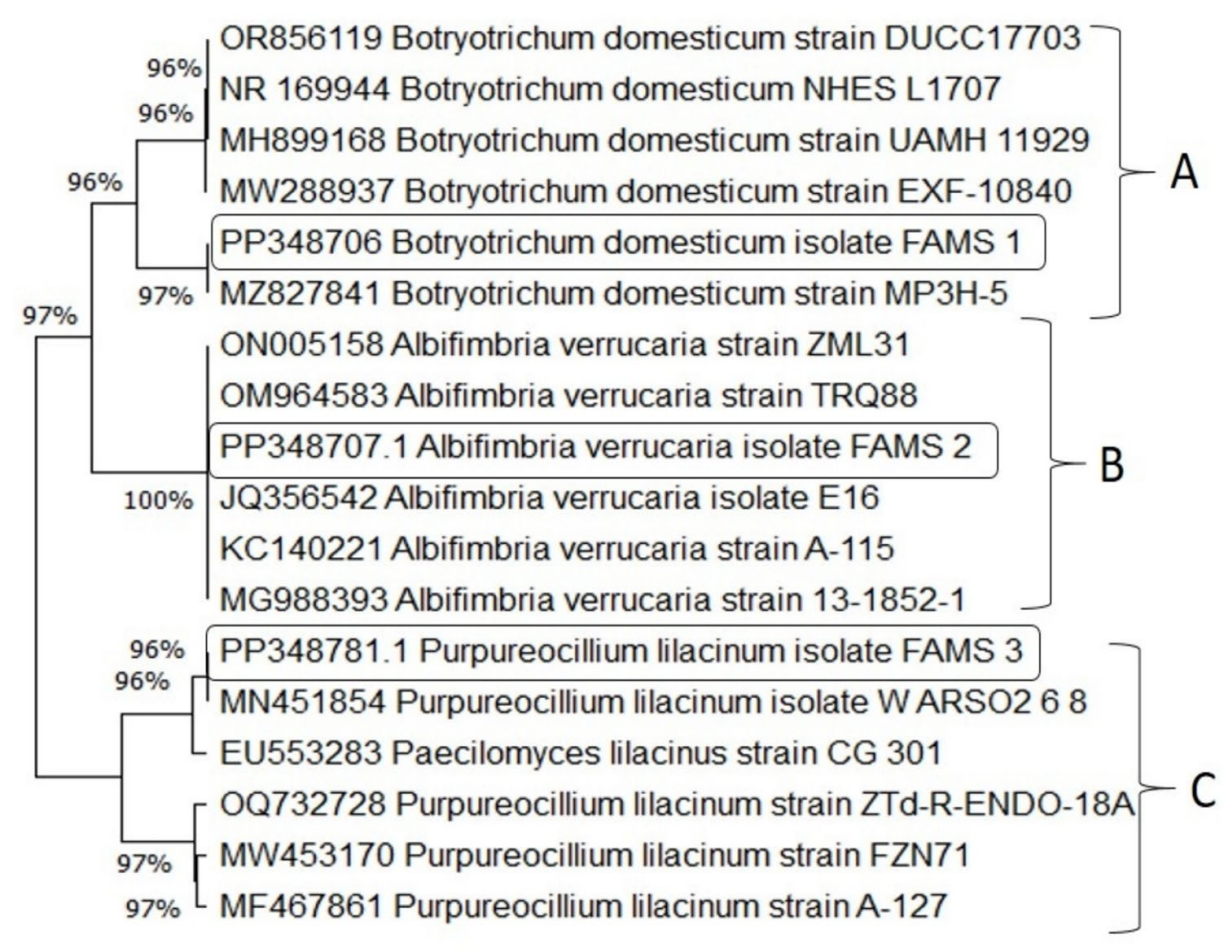
Phylogenetic tree based on ITS rDNA sequence analysis of the three fungal isolates and their closest matches retrieved from the NCBI GenBank database. Sequences were aligned and analyzed using the Neighbor-Joining method in MEGA11 with 1000 bootstrap replications. The isolates FAMS 1, FAMS 2, and FAMS 3 clustered in distinct clades (**A**, **B**, and **C**, respectively), each supported by high bootstrap values

Phylogenetic analysis was performed using MEGA11 software based on the aligned ITS rDNA sequences. The top five homologous sequences for each isolate (95–100% identity) were used to construct the phylogenetic tree (Fig. 2), which revealed three distinct clades: Group A (FAMS 1), Group B (FAMS 2), and Group C (FAMS 3). Group B exhibited the highest DNA sequence identity and similarity among the three.

### Effect of fungal isolates on the second-instar larvae of *S. frugiperda*

As shown in Table 1, second-instar larvae of *S. frugiperda* were highly susceptible to infection by *A. verrucaria* (isolate 2), which resulted in a mortality rate of 75.55% at 3 days post-treatment when applied at 1 × 10^9^ conidia mL⁻^1^. After 14 days, *A. verrucaria* caused 84.44% mortality, followed by *B. domesticum* (isolate 1) and *P. lilacinum* (isolate 3), each with 60% mortality at the same concentration. In contrast, the control group showed only 3.33% mortality. Consistent with the mortality data, *A. verrucaria* (isolate 2) exhibited the lowest median concentration (LC_50_) of 2 × 10^7^ conidia mL⁻^1^and a quarter lethal concentration (LC_25_) of 1 × 10^6^ conidia mL⁻^1^, indicating higher virulence compared to *B. domesticum* (LC_50_ = 1 × 10^8^ conidia mL⁻^1^, LC_25_ = 9 × 10^6^ conidia mL⁻^1^) (Table S1). When *S. frugiperda* larvae were treated with *B. domesticum*, *A. verrucaria*, and *P. lilacinum* at concentrations of 1 × 10^8^ and 1 × 10^9^ conidia mL⁻^1^, *A. verrucaria* (isolate 2) demonstrated the shortest median lethal time (LT_50_) values of (7.3 and 0.46 days), and the shortest quarter lethal time (LT_25_) values of (0.6 and 0.055 days), respectively (Table S2).

**Table 1:** Mean cumulative mortality (% ± SD) of the second-instar larvae of *S. frugiperda* treated with different concentrations of indigenous isolates of the entomopathogenic fungi *Botryotrichum domesticum*, *Albifimbria verrucaria*, and *Purpureocillium lilacinum* under laboratory conditions

Treatments	Concentration (conidia mL⁻^1^)	Days post-treatment					
		3 days	5 days	7 days	9 days	12 days	14 days
*B. domesticum*	10^6^	7.77 ± 1.92^cd^	10 ± 3.33^c^	13.33 ± 5.77^de^	13.33 ± 5.77^de^	13.33 ± 5.77^fg^	13.33 ± 5.77^ef^
	10^7^	12.22 ± 6.94^c^	13.33 ± 5.77^c^	23.33 ± 5.77^cde^	23.33 ± 5.77^cde^	23.33 ± 5.77^defg^	23.33 ± 5.77^de^
	10^8^	13.33 ± 5.77^c^	20 ± 0^c^	25.55 ± 5.09^cde^	33.33 ± 6.67^bcd^	40 ± 11.54^bcde^	62.22 ± 3.84^b^
	10^9^	14.44 ± 5.09^c^	22.22 ± 3.84^c^	30 ± 10^bcd^	40 ± 13.33^bc^	51.11 ± 16.77^bc^	60 ± 13.33^b^
*A. verrucaria*	10^6^	10 ± 0^cd^	16.67 ± 5.77^c^	23.33 ± 11.54^cde^	23.33 ± 11.54^cde^	26.67 ± 5.77^cdef^	26.67 ± 5.77^cde^
	10^7^	13.33 ± 5.77^c^	20 ± 17.32^c^	30 ± 17.32^bcd^	33.33 ± 11.54^bcd^	40 ± 0b^cde^	46.67 ± 11.54^bc^
	10^8^	40 ± 0^b^	46.67 ± 5.77^b^	53.33 ± 5.77^b^	53.33 ± 5.77^b^	53.33 ± 5.77^b^	53.33 ± 5.77^b^
	10^9^	75.55 ± 3.8^a^	80 ± 11.54^a^	82.22 ± 10.18^a^	82.22 ± 10.18^a^	82.22 ± 10.18^a^	84.44 ± 7.69^a^
*P. lilacinum*	10^6^	10 ± 0^cd^	16.67 ± 11.54^c^	16.67 ± 11.54^de^	16.67 ± 11.54^cde^	16.67 ± 11.54^efg^	20 ± 10^def^
	10^7^	10 ± 0^cd^	16.67 ± 5.77^c^	31.11 ± 7.7^bcd^	36.67 ± 5.77^bcd^	36.67 ± 5.77^bcdef^	40 ± 10^bcd^
	10^8^	10 ± 0^cd^	21.11 ± 8.39^c^	33.33 ± 5.77^bcd^	38.89 ± 8.38^bcd^	44.44 ± 10.18^bcd^	55.55 ± 7.7^b^
	10^9^	13.33 ± 5.77^c^	23.33 ± 5.77^bc^	44.44 ± 3.84^bc^	51.11 ± 3.85^b^	53.33 ± 6.67^b^	60 ± 0b
Control		0^d^	0^c^	0^e^	3.33 + 5.77^e^	3.33 + 5.77^g^	3.33 + 5.77^f^
*P*-value		0.001	0.001	0.001	0.001	0.001	0.001
*F*-value (df)		77.45 (12, 26)	18.56 (12, 26)	15.98(12, 26)	16.46 (12, 26)	18.45 (12, 26)	28.62 (12, 26)

*Means sharing the same letter within the same column are not significantly different at *p* < 0.05 according to Tukey’s test

### Effect of fungal isolates on *S. frugiperda* Pupae

Pupal mortality rates following treatment with *B. domesticum* (isolate 1), *A. verrucaria* (isolate 2), and *P. lilacinum* (isolate 3) at two concentrations (1 × 10^6^ and 1 × 10^7^ conidia mL⁻^1^) are presented in Table 2. At 1 × 10^7^ conidia mL⁻^1^, mortality after 3 days was 26.66% for *B. domesticum*, 20% for *P. lilacinum*, and 13.33% for *A. verrucaria*. However, by day 7, *P. lilacinum* and *B. domesticum* induced significant mortality rates of 80% and 46.66%, respectively, with no mortality recorded in the control.

**Table 2:** Cumulative mortality (mean ± SD) percentage of *S. frugiperda* pupae infected with two concentrations of *Botryotrichum domesticum*, *Albifimbria verrucaria*, and *Purpureocillium lilacinum* under laboratory conditions

Treatment	Concentration (conidia mL⁻^1^)	Days post-treatment		
		3 days	5 days	7 days
*B. domesticum*	10^6^	13.33 ± 9.42^a^	20 ± 0^ab^	33.33 ± 9.42^bc^
	10^7^	26.66 ± 18.85^a^	40 ± 16.32^ab^	46.66 ± 9.42^bc^
*A. verrucaria*	10^6^	6.66 ± 9.42^a^	20 ± 0^ab^	26.66 ± 9.42^cd^
	10^7^	13.33 ± 9.42^a^	26.66 ± 9.42^ab^	33.33 ± 9.42^bc^
*P. lilacinum*	10^6^	13.33 ± 18.85^a^	20 ± 16.32^ab^	60 ± 0^ab^
	10^7^	20 ± 16.32^a^	53.33 ± 18.85^a^	80 ± 16.32^a^
Control		0^a^	0 b	0^d^
*P-*value		0.564	0.014	0.001
*F-*value (df)		0.83 (6, 14)	4.12 (6, 14)	14.67 (6, 14)

*Means sharing the same letter within the same column are not significantly different at* p* < 0.05 according to Tukey’s test

### Effect of the fungal metabolites on *S. frugiperda*

As shown in Table 3, second-instar larvae of *S. frugiperda* exhibited notable susceptibility to the crude fungal metabolites, particularly those of *P. lilacinum* (isolate 3), which induced a mortality rate of 36.67% at 3 days post-treatment. The highest larval mortality (50%) was observed at 14 days post-treatment in larvae treated with the crude metabolites of *B. domesticum* (isolate 1), followed by 43.33% for *P. lilacinum* (isolate 3), and 13.33% for *A. verrucaria* (isolate 2). In contrast, the control group exhibited only 3.33% mortality.

**Table 3:** Mean cumulative mortality (% ± SD) of second-instar larvae of *S. frugiperda* treated with crude secondary metabolites of the entomopathogenic fungi *Botryotrichum domesticum*, *Albifimbria verrucaria*, and *Purpureocillium lilacinum* under laboratory conditions

Treatments	Days post-treatment					
	3 days	5 days	7 days	9 days	12 days	14 days
*B. domesticum*	26.67 ± 5.77^ab^	30 ± 0^a^	30 ± 0^a^	43.33 ± 15.27^a^	43.33 ± 15.27^a^	50 ± 17.32^a^
*A. verrucaria*	10 ± 0^bc^	13.33 ± 5.77^b^	13.33 ± 5.77^b^	13.33 ± 5.77^b^	13.33 ± 5.77^b^	13.33 ± 5.77^b^
*P. lilacinum*	36.67 ± 11.55^a^	40 ± 10^a^	40 ± 10^a^	43.33 ± 5.77^a^	43.33 ± 5.77^a^	43.33 ± 5.77^a^
Control	0^c^	0^b^	0^b^	0b	3.33 ± 5.77^b^	3.33 ± 5.77^b^
*P*-value	0.0001	0.001	0.001	0.001	0.001	0.001
*F*-value (df)	19.47(3, 8)	28.25(3, 8)	28.25(3, 8)	19.11(3, 8)	15.30(3, 8)	18.67(3, 8)

*Means sharing the same letter within the same column are not significantly different at *P* < 0.05 according to Tukey’s test

### Chemical composition of the secondary metabolites of the three isolated EPF

The chemical constituents of the secondary metabolites from the three isolated EPF were identified using GC-MS. For *B. domesticum* (isolate 1), the 5th major components were n-oleic acid (25.99%), n-hexadecanoic acid (11.89%), 9,12-octadecadienoic acid (Z,Z) (8.31%), erucic acid (5.44%), and glycidyl oleate (5.05%) (Table S3) and (Table 4). For *A. verrucaria* (isolate 2), the dominant compounds included n-oleic acid (19%), n-hexadecanoic acid (10.91%), cis-13-docosenoyl chloride (7.68%), 9,12-octadecadienoic acid (Z,Z) (6.32%), and glycidyl oleate (5.79%) (Table S4) and (Table 4). For *P. lilacinum* (isolate 3), the main identified constituents were oleic acid (16.57%), n-hexadecanoic acid (10.58%), cis-13-docosenoyl chloride (9.46%), glycidyl oleate (7.18%), and 9-octadecenoic acid (Z)-,2,3-dihydroxypropyl ester (5.53%) (Table S5) and (Table 4).

**Table 4:** Major compounds in the secondary metabolites of *Botryotrichum domesticum*, *Albifimbria verrucaria*, and *Purpureocillium lilacinum* identified by gas chromatography-mass spectrometry (GC-MS)

Isolate	*RT (min)	Compounds	Area%	**MF	Molecular formula
*B. domesticum*	26.45	n-Hexadecanoic acid	11.89	923	C_16_H_32_O_2_
	29.47	9,12-Octadecadienoic acid-(Z,Z)	8.31	925	C_18_H_32_O_2_
	29.68	Oleic acid	25.99	930	C18H_34_O_2_
	40.11	Erucic acid	5.44	846	C_22_H_42_O_2_
	40.59	Glycidyl oleate	5.05	865	C_21_H_38_O_3_
*A. verrucaria*	26.45	n-Hexadecanoic acid	10.91	919	C_16_H_32_O_2_
	29.46	9,12-Octadecadienoic acid-(Z,Z)	6.32	907	C_18_H_32_O_2_
	29.67	Oleic acid	19.00	920	C_18_H_34_O_2_
	34.89	Glycidyl oleate	5.79	902	C_21_H_38_O_3_
	40.11	cis-13-Docosenoyl chloride	7.68	841	C_22_H_41_C_lO_
*P. lilacinum*	26.4444	n-Hexadecanoic acid	10.5858	918	C_16_H_32_O2
	29.6767	Oleic acid	16.5757	916	C_18_H_34_O_2_
	34.1414	9-Octadecenoic acid (Z)-, 2,3-dihydroxypropyl ester	5.5353	871	C_21_H_40_O_4_
	34.8989	Glycidyl oleate	7.1818	902	C_21_H_38_O_3_
	40.11	cis-13-Docosenoyl chloride	9.46	850	C_22_H_41_C_lO_

^*^RT, retention time; **Mf, match factor

### Effect of isolated entomopathogenic fungi and their metabolites on oxidative stress enzymes

**Fig. 3:**
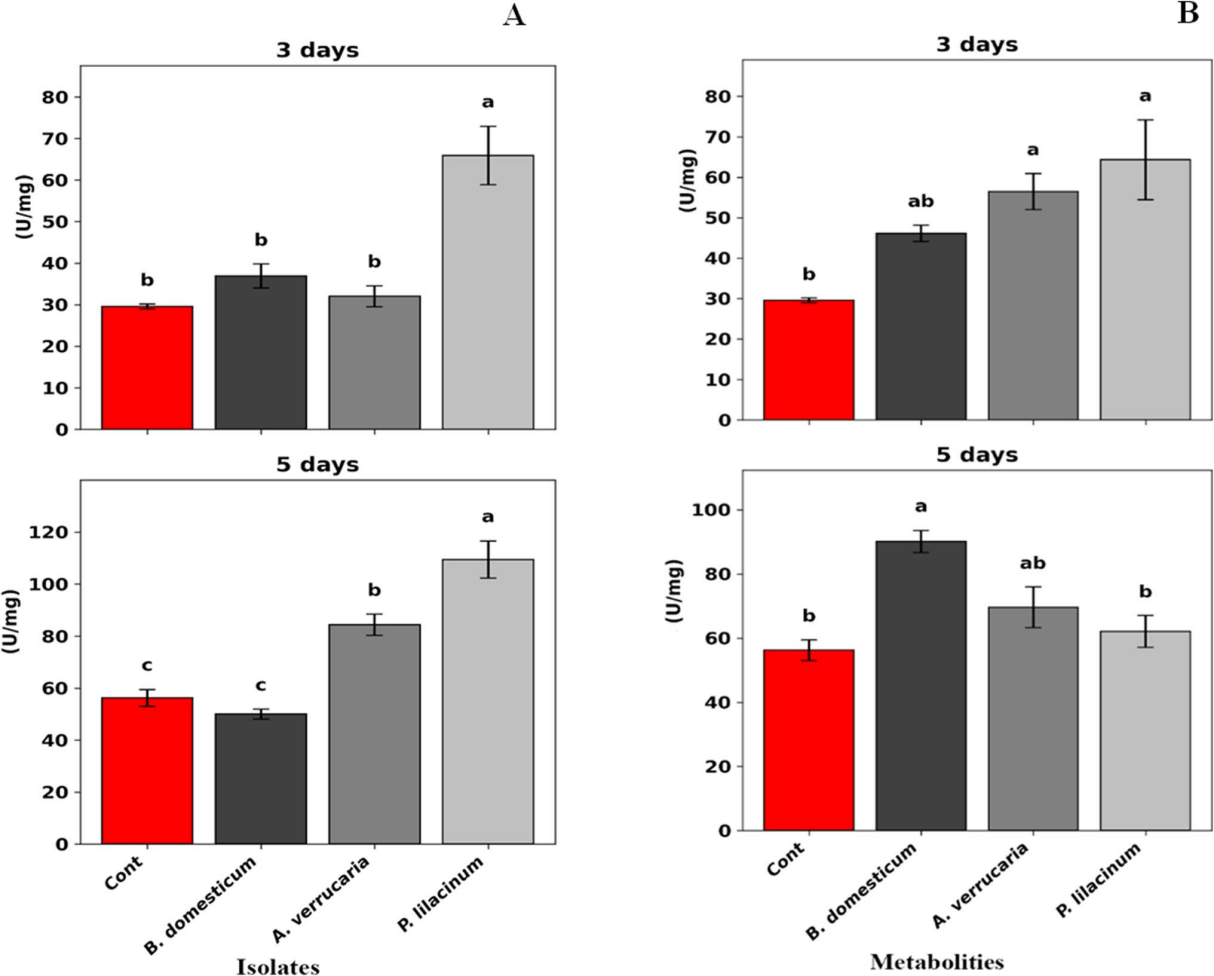
Catalase (CAT) enzyme activity in second-instar larvae of *Spodoptera frugiperda* following treatment with **A** conidial suspensions (1 × 10^9^ conidia mL⁻^1^) and **B** crude secondary metabolites of *Botryotrichum domesticum*, *Albifimbria verrucaria*, and *Purpureocillium lilacinum*. Measurements were taken at 3 and 5 days post-treatment. Bars represent mean enzyme activity (U/mg protein)

As shown in Fig. 3 and Table S6, catalase (CAT) activity in *S. frugiperda* larvae significantly increased following treatment with the fungal isolates. At 3 days post-treatment, *P. lilacinum* (isolate 3) induced the highest CAT activity, reaching 65.92 U/mg of protein (*F* = 17.70, *P* = 0.001). This increase was more pronounced after 5 days, with CAT activity rising to 109.45 U/mg of protein (*F* = 36.93, *P* = 0.001), compared to the control (56.22 U/mg of protein). Regarding the crude fungal metabolites, a slight but statistically significant variation in CAT activity was observed among the three isolates at 3 days post-treatment (*F* = 7.42, *P* = 0.011). After 5 days, *B. domesticum* (isolate 1) showed the highest CAT activity (90.12 U/mg of protein), which was significantly higher than the control (*F* = 10.15, *P* = 0.004) (Fig. 3) and (Table S7).

## Discussion

Several control strategies have been developed to manage *S. frugiperda*, including chemical pesticides, biological control agents (e.g., predatory and parasitic organisms and microbial-based insecticides), and botanical pesticides (Midega et al. 2018; Kassie et al. 2020). Among these, microbial insecticides have long played an integral role in IPM programs (Souza et al. 2019). Various microbial agents including EPF, bacteria, viruses, and nematodes are known to target *S. frugiperda* (Guo et al. 2020). This study focused on the isolation and identification of indigenous EPF from local soils and the evaluation of their insecticidal activity against *S. frugiperda*.

The EPF are commonly found within the phylum Zygomycota, Ascomycota, and Deuteromycota (Shah and Pell 2003), with most pathogenic species in Ascomycota exhibiting a well-developed parasitic phase capable of invading the insect host (Araújo and Hughes 2016). Based on morphological and molecular identification, the isolated strains were identified as *B. domesticum* isolate MP3H-5, *A. verrucaria* isolate E16, and *P. lilacinum* isolate WARSO2 6 8 all belonging to the phylum Ascomycota (Dong et al. 2012; Schultes et al. 2019; Weaver et al. 2021).

Among the tested isolates, *A. verrucaria* (E16) showed the highest virulence, inducing up to 84.88% larval mortality and recording the lowest LC_50_ (2 × 10^7^ conidia mL⁻^1^) and LT_50_ (0.46 days). This is consistent with the findings of Assaf (2020), who reported that *A. verrucaria* caused 87.5% mortality in first instar larvae and 31.45% in fourth instar larvae of *Epilachna chrysomelina*, 3 days post-treatment. Similarly, Chandele et al. (2020) reported that enzymes from *A. verrucaria*, when combined with *Metarhizium anisopliae*, successfully controlled thrips and jassids, achieving mortality rates of 51.75% and 38.4%, respectively. Both *B. domesticum* (MP3H-5) and *P. lilacinum* (W ARSO2 6 8) resulted in 60% larval mortality. Previous studies have shown that the genus *Botryotrichum* produces lipolytic enzymes responsible for its toxicity (Moharram et al. 2021). Abdel Galil et al. (2019) found that *Botryotrichum atrogriseum* caused 30–50% mortality in bean aphids within 4 days. *Purpureocillium lilacinum*, widely distributed in soil, acts as both an insect parasite and a biocontrol agent against phytopathogenic fungi and bacteria, while also promoting plant growth (Moreno-Gavíra et al. 2020). Interestingly, *P. lilacinum* displayed a different infection pattern, especially at the pupal stage, where it recorded the highest pupal mortality of 80% within 7 days after treatment at 1 × 10^7^ conidia mL⁻^1^. These findings align with Liu et al. (2022a, b), who found that *P. lilacinum* caused 90% mortality in *S. frugiperda* larvae within 108 h post-treatment.

Secondary metabolites play a key role in EPF pathogenicity due to their bioactive properties (Pedrini 2022; Toopaang et al. 2023). Therefore, GC-MS analysis was employed to identify the major compounds in the crude extracts. In this study, oleic acid was found to be the predominant compound in the extracts of *B. domesticum*, followed by *A. verrucaria* and *P. lilacinum*. Oleic acid is a known insecticidal compound with contact toxicity reported against whiteflies, mosquitoes, and beetles (Don‐Pedro 1990; Ramsewak et al. 2001; Lee et al. 2013; Hadi and Sabit 2023). In the current study, crude extract of *B. domesticum* caused 50% mortality in *S. frugiperda* larvae after 14 days, while extracts of *P. lilacinum* and *A. verrucaria* induced 43.33% and 13.33% mortality, respectively, compared to 3.33% in the control group 14 days after treatment. Chen and Hu (2021) reported that secondary metabolites of *P. lilacinum* exhibit diverse biological activities, including anticancer, antibacterial, and insecticidal effects. Similarly, Woo et al. (2020) demonstrated that metabolites from *Lecanicillium attenuatum* had strong insecticidal and growth-regulating properties, with mortality exceeding 90% in third instar larvae of *Aedes albopictus* and 70% in *Plutella xylostella*. Ridaoui et al. (2022) reported that the antioxidant system is a vital component of insect defense, helping to regulate ROS and prevent oxidative damage; enzymes such as CAT play synergistic roles in detoxification pathways. In this study, CAT activity increased significantly in second-instar larvae of *S. frugiperda* treated with *P. lilacinum* (isolate 3), showing a 2.2-fold increase after 3 days. After 5 days of treatment, CAT activity reached 84.32 and 109.45 U/mg protein in larvae treated with *A. verrucaria* and *P. lilacinum*, respectively, reflecting 1.4- and 1.9-fold increases compared to the control. Moreover, CAT activity was significantly elevated in larvae treated with crude fungal metabolites of *A. verrucaria* and *P. lilacinum* at 3 days post-treatment. At 5 days post-treatment, *B. domesticum* (isolate 1) induced a 1.6-fold increase in CAT activity compared to the control, indicating elevated oxidative stress and hydrogen peroxide accumulation. Similarly, Abarna et al. (2024) observed enhanced antioxidant enzyme activity in *P. xylostella* larvae 4 days after treatment with *M. anisopliae*. However, the level of enzyme induction may vary depending on the fungal isolate (Naeem et al. 2020).

To conclude, this study is the first to evaluate the insecticidal activity of *B. domesticum*, *A. verrucaria*, and *P. lilacinum* against *S. frugiperda* in Egypt. The findings demonstrate that the indigenous fungal isolates and their secondary metabolites exhibit significant virulence against both larval and pupal stages of *S. frugiperda*. Among them, *A. verrucaria* stood out by inducing up to 80% mortality in larvae, supporting its potential as a promising biocontrol agent. Future studies, including field trials, are necessary to assess the impact of UV radiation and other biotic and abiotic factors on fungal efficacy. These results provide a valuable foundation for the development of microbial biocontrol strategies for managing *S. frugiperda*.

## Supplementary Information

Below is the link to the electronic supplementary material.

ESM 1 (DOCX 230 KB)

## Data Availability

The datasets generated during the current study are available from the corresponding author upon reasonable request.
