# Supplementary material for: Isolation, Identification, and Pathogenicity of Entomopathogenic Fungal Strains and Their Secondary Metabolites Against Spodoptera frugiperda (Lepidoptera: Noctuidae)
Source: Neotrop Entomol. 2026 Feb 18;55(1):11. doi: 10.1007/s13744-026-01360-3 (PMC12916973; doi:10.1007/s13744-026-01360-3)
Supplement: Supplementary file 1 — (DOCX 230 KB) [file 13744_2026_1360_MOESM1_ESM.docx]

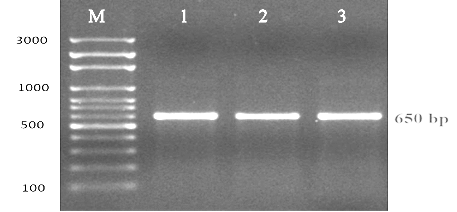


**Fig S1.** PCR amplification of DNA extracted from the three fungal isolates: Lane M, 100 bp DNA ladder. Lane 1, 2, & 3: 650 bp ITS rDNA regions for the three isolates.

**Table S1.** Lethal concentration (LC_25_ and LC_50_) values of *Botryotrichum domesticum,* *Albifimbria verrucaria* and *Purpureocillium lilacinum* against the 2^nd^ instar larvae of *S. frugiperda* under laboratory conditions.

| **Treatment** | **LC_25_ (conidia mL⁻¹)**  **(95% confidence limits)** | **LC_50_ (conidia mL⁻¹)**  **(95% confidence limits)** | **Slope± SE** | ***X^2^*** |
| --- | --- | --- | --- | --- |
|  |  |  |  |  |
| ***Botryotrichum domesticum*** | 9 х10^6^  (2х10^6^- 2 х 10^7^) | 1 х10^8^  (6 х 10^7^- 4 х10^8^) | 0.542±0.0912 | 4.8839 |
| ***Albifimbria verrucaria*** | 1х10^6^  (1 х 10^5^ -3 х10^6^) | 2х10^7^  (1 х 10^7^- 6 х10^7^) | 0.5122±0.0875 | 2.9897 |
| ***Purpureocillium lilacinum*** | 2 х10^6^  (1 х10^5^ - 7 х10^6^) | 1 х10^8^  (4 х10^7^ - 6 х10^8^) | 0.3795±0.0845 | 1.9815 |

* *X^2^.* Chi square

| **Treatments** | **Concentration (conidia mL⁻¹)** | **LT_25_ (days)**  **(95% confidence limits)** | **LT_50_ (days)**  **(95% confidence limits)** | **Slope± SE** | ***χ^2^*** |
| --- | --- | --- | --- | --- | --- |
|  |  |  |  |  |  |
| ***Botryotrichum domesticum*** | 1 х10^6^ | 95.4 | 2762.9 | 0.46±0.30 | 0.57 |
|  | 1 х 10^7^ | 13.1 | 110.8 | 0.72±0.26 | 2.38 |
|  | 1х10^8^ | 5.9  (4.5-7.2) | 13.3  (10.8-19.4) | 1.94±0.37 | 4.18 |
|  | 1х10^9^ | 5.3  (3.9-6.4) | 11.6  (9.6-15.5) | 1.99±0.36 | 0.79 |
| ***Albifimbria verrucaria*** | 1 х10^6^ | 10.5  (7.9-17.8) | 53.9  (26.3-597.6) | 0.94±0.26 | 1.23 |
|  | 1 х 10^7^ | 6  (4.7-7) | 16.7  (13.2-25.3) | 1.51±0.25 | 0.44 |
|  | 1х10^8^ | 0.6  (0.02-1.5) | 7.3  (7.5-4.49) | 0.61±0.18 | 0.72 |
|  | 1х10^9^ | 0.055  (0.0003-0.27) | 0.46  (0.03-1.15) | 0.41±0.36 | 0.15 |
| ***Purpureocillium lilacinum*** | 1 х10^6^ | 33.9 | 848.6 | 0.48±0.27 | 1.21 |
|  | 1 х 10^7^ | 6.6  (5.4-7.8) | 18  (14.1-28.2) | 1.55±0.26 | 3.61 |
|  | 1х10^8^ | 5.8  (4.3-6.9) | 12.5  (10.3-17.5) | 1.99±0.36 | 0.60 |
|  | 1х10^9^ | 4.6  (3.3-5.6) | 9.9  (8.3-12.4) | 2.04±0.35 | 2.01 |

**Table S2**. Lethal time (LT₂₅ and LT₅₀) values of the entomopathogenic fungi *Botryotrichum domesticum,* *Albifimbria verrucaria* and *Purpureocillium lilacinum* against the 2^nd^ instar larvae of *S. frugiperda* under laboratory conditions.

**Table S3.** Major chemical compounds of *Botryotrichum domesticum* secondary metabolites as identified using GC–MS. Compounds are listed in order of increasing retention time (RT).

| ***Botryotrichum domesticum*** | | | |
| --- | --- | --- | --- |
| ***RT** | **Area %** | **Compound Name** | ****MF** |
| 26.45 | 11.89 | n-Hexadecanoic acid | 923 |
| 28.21 | 0.89 | HEXADECANOIC ACID, TRIMETHYLSILYL ESTER | 884 |
| 28.69 | 0.84 | 9-OCTADECENOIC ACID (Z)- | 832 |
| 29.47 | 8.31 | 9,12-Octadecadienoic acid (Z,Z)- | 925 |
| 29.68 | 25.99 | Oleic Acid | 930 |
| 30.12 | 3.53 | OCTADECANOIC ACID | 859 |
| 31.32 | 1.60 | Isopropyl linoleate | 819 |
| 31.47 | 1.48 | trans-13-Octadecenoic acid | 816 |
| 32.09 | 1.17 | Glycidyl oleate | 821 |
| 32.98 | 1.70 | cis-13-Eicosenoic acid | 896 |
| 34.00 | 3.29 | 9,12-Octadecadienoic acid (Z,Z)-, 2-hydroxy-1-(hydroxymethyl)ethyl ester | 888 |
| 34.14 | 3.62 | 9-OCTADECENOIC ACID (Z)-, 2-HYDROXY-1-(HYDROXYMET HYL)ETHYL ESTER | 865 |
| 34.24 | 0.78 | 9-OCTADECENOIC ACID (Z)-, 2-HYDROXY-1-(HYDROXYMET HYL)ETHYL ESTER | 840 |
| 34.75 | 2.67 | 9,12,15-Octadecatrienoic acid, 2,3-dihydroxypropyl ester, (Z,Z,Z)- | 832 |
| 34.89 | 4.40 | Glycidyl oleate | 907 |
| 34.98 | 0.92 | Glycidyl oleate | 841 |
| 35.59 | 1.19 | Methyl 11-docosenoate | 878 |
| 36.18 | 2.32 | Erucic acid | 898 |
| 37.26 | 0.80 | cis-13-Eicosenoic acid | 851 |
| 37.81 | 2.40 | Erucic acid | 825 |
| 37.98 | 1.37 | Glycidyl oleate | 854 |
| 40.04 | 0.74 | OLEIC ACID, 3-(OCTADECYLOXY)PROPYL ESTER | 791 |
| 40.11 | 5.44 | Erucic acid | 846 |
| 40.59 | 5.05 | Glycidyl oleate | 865 |
| 41.91 | 3.36 | ISOCHIAPIN B %2 | 804 |
| 42.42 | 1.16 | Glycidyl oleate | 799 |
| 42.58 | 0.88 | 2-HYDROXY-3-[(9E)-9-OCTADEC ENOYLOXY]PROPYL (9E)-9-OCTADECENOATE | 754 |
| 43.87 | 0.95 | OLEIC ACID, 3-(OCTADECYLOXY)PROPYL ESTER | 804 |
| 45.06 | 1.29 | 2-HYDROXY-3-[(9E)-9-OCTADEC ENOYLOXY]PROPYL (9E)-9-OCTADECENOATE | 784 |

*RT= retention time; **Mf= match factor

**Table S4.** Major chemical compounds of *Albifimbria verrucaria* secondary metabolites as identified using GC–MS. Compounds are listed in order of increasing retention time (RT).

| ***Albifimbria verrucaria*** | | | |
| --- | --- | --- | --- |
| ***RT** | **Area %** | **Compound Name** | ****MF** |
| 4.30 | 0.84 | BICYCLO[3.1.1]HEPTANE, 6,6-DIMETHYL-2-METHYLENE-, (1S)- | 870 |
| 25.69 | 1.62 | HEXADECANOIC ACID, METHYL ESTER | 917 |
| 26.45 | 10.91 | n-Hexadecanoic acid | 919 |
| 28.21 | 1.23 | Palmitic Acid, TMS derivative | 848 |
| 28.69 | 4.09 | Methyl 9-cis,11-trans-octadecadienoate | 909 |
| 28.87 | 3.03 | 11-Octadecenoic acid, methyl ester | 909 |
| 29.46 | 6.32 | 9,12-Octadecadienoic acid (Z,Z)- | 907 |
| 29.67 | 19.00 | Oleic Acid | 920 |
| 30.11 | 2.07 | Oleic Acid | 863 |
| 31.15 | 0.56 | 9-OCTADECENOIC ACID (Z)- | 809 |
| 31.32 | 1.92 | Isopropyl linoleate | 830 |
| 31.47 | 1.85 | trans-13-Octadecenoic acid | 823 |
| 32.09 | 1.45 | Erucic acid | 810 |
| 33.99 | 4.01 | 9,12-Octadecadienoic acid (Z,Z)-, 2-hydroxy-1-(hydroxymethyl)ethyl ester | 888 |
| 34.14 | 4.64 | 9-Octadecenoic acid (Z)-, 2,3-dihydroxypropyl ester | 867 |
| 34.24 | 0.97 | 9-OCTADECENOIC ACID (Z)-2-hydroxy-1-(hydroxymethyl)ethyl ester | 836 |
| 34.75 | 3.37 | 9,12,15-OCTADECATRIENOIC ACID, 2,3-DIHYDROXYPROPYL ESTER, (Z,Z,Z)- | 838 |
| 34.89 | 5.79 | Glycidyl oleate | 902 |
| 34.98 | 1.16 | Glycidyl oleate | 842 |
| 35.25 | 0.45 | 9-OCTADECENOIC ACID (Z)- | 841 |
| 35.37 | 0.79 | Erucic acid | 828 |
| 35.58 | 1.36 | Erucic acid | 874 |
| 36.17 | 0.80 | cis-13-Eicosenoic acid | 839 |
| 37.26 | 0.92 | cis-13-Eicosenoic acid | 853 |
| 37.65 | 0.50 | n-Propyl 11-octadecenoate | 846 |
| 37.81 | 2.93 | Erucic acid | 823 |
| 37.98 | 1.63 | Glycidyl oleate | 840 |
| 40.11 | 7.68 | cis-13-Docosenoyl chloride | 841 |
| 40.40 | 0.61 | 9-Octadecenoic acid (Z)-, 2-hydroxy-1-(hydroxymethyl)ethyl ester | 814 |
| 40.58 | 4.86 | Glycidyl oleate | 863 |
| 42.42 | 1.48 | Glycidyl oleate | 803 |
| 42.58 | 1.15 | STIGMAST-5-EN-3-OL, (3á,24S)- | 756 |

*RT= retention time; **Mf= match factor

**Table S5.** Major chemical compounds of *Purpureocillium lilacinum* secondary metabolites as identified using GC–MS. Compounds are listed in order of increasing retention time (RT).

| ***Purpureocillium lilacinum*** | | | |
| --- | --- | --- | --- |
| **RT** | **Area %** | **Compound Name** | ****MF** |
| 4.2929 | 1.1818 | Bicyclo[3.1.1]heptane, 6,6-dimethyl-2-methylene-, (1S)- | 900 |
| 26.4444 | 10.5858 | n-Hexadecanoic acid | 918 |
| 28.2121 | 1.5252 | Palmitic Acid, TMS derivative | 847 |
| 28.7070 | 1.1717 | 9-OCTADECENOIC ACID (Z)- | 834 |
| 28.8787 | 0.6767 | trans-13-Octadecenoic acid | 845 |
| 29.4646 | 5.0202 | 9,12-Octadecadienoic acid (Z,Z)- | 902 |
| 29.6767 | 16.5757 | Oleic Acid | 916 |
| 30.1111 | 1.3030 | Oleic Acid | 869 |
| 31.1616 | 0.7070 | 9-OCTADECENOIC ACID (Z)- | 822 |
| 31.3232 | 2.2929 | Isopropyl linoleate | 824 |
| 31.4848 | 2.1717 | trans-13-Octadecenoic acid | 823 |
| 32.0909 | 1.8989 | Glycidyl oleate | 823 |
| 34.0000 | 4.8686 | 9,12-Octadecadienoic acid (Z,Z)-, 2-hydroxy-1-(hydroxymethyl)ethyl ester | 885 |
| 34.1414 | 5.5353 | 9-Octadecenoic acid (Z)-, 2,3-dihydroxypropyl ester | 871 |
| 34.2424 | 1.1313 | 9-OCTADECENOIC ACID (Z)- | 830 |
| 34.7575 | 4.1515 | (9E,12E)-9,12-OCTADECADIENO YL CHLORIDE # | 851 |
| 34.8989 | 7.1818 | Glycidyl oleate | 902 |
| 34.9898 | 1.4242 | 9-OCTADECENOIC ACID (Z)- | 827 |
| 35.2525 | 0.5959 | 9-OCTADECENOIC ACID (Z)- | 839 |
| 35.3737 | 1.0505 | Erucic acid | 828 |
| 35.58 | 1.59 | Erucic acid | 875 |
| 37.26 | 1.11 | cis-13-Eicosenoic acid | 840 |
| 37.65 | 0.96 | 9-Octadecenoic acid (Z)-, 2-hydroxy-1-(hydroxymethyl)ethyl ester | 832 |
| 37.81 | 4.21 | Erucic acid | 826 |
| 37.97 | 1.51 | Glycidyl oleate | 840 |
| 40.11 | 9.46 | cis-13-Docosenoyl chloride | 850 |
| 40.41 | 0.91 | 2-HYDROXY-3-[(9E)-9-OCTADEC ENOYLOXY]PROPYL (9E)-9-OCTADECENOATE # | 791 |
| 40.59 | 6.30 | Glycidyl oleate | 865 |
| 42.42 | 1.75 | 2-HYDROXY-3-[(9E)-9-OCTADEC ENOYLOXY]PROPYL (9E)-9-OCTADECENOATE # | 774 |
| 42.58 | 1.21 | STIGMAST-5-EN-3-OL, (3á,24S)- | 753 |

*RT= retention time; **Mf= match factor

**Table (S6):** Catalase enzyme (CAT) activity (mean ± SD) in the 2^nd^ instar larvae of *Spodoptera frugiperda* after 3 and 5 days of treatment with conidial suspensions (1 × 10⁹ conidia mL⁻¹) of *Botryotrichum domesticum*, *Albifimbria verrucaria*, and *Purpureocillium lilacinum*.

| Treatment | 3 days | 5 days |
| --- | --- | --- |
| *Botryotrichum domesticum* | 36.9±4.05b | 49.98±2.66c |
| *Albifimbria verrucaria* | 32±3.55b | 84.31±5.77b |
| *Purpureocillium lilacinum* | 65.92±9.91a | 109.44±10.08a |
| Control | 29.55±0.8b | 56.21±4.52c |
| *P*-value | 0.001 | 0.001 |
| *F*-value (df) | 17.70 (3,8) | 36.93 (3,8) |

Values are presented as mean ± SD. Within each column, means followed by different superscript letters are significantly different (ANOVA followed by Tukey’s HSD test, p < 0.05).

**Table (S7):** Catalase enzyme (CAT) activity (mean ± SD) in the 2^nd^ instar larvae of *Spodoptera frugiperda* exposed to secondary metabolites of *Botryotrichum domesticum*, *Albifimbria verrucaria*, and *Purpureocillium lilacinum*.

| Treatment | 3 days | 5 days |
| --- | --- | --- |
| *Botryotrichum domesticum* metabolites | 46.09±2.86ab | 90.11±4.84a |
| *Albifimbria verrucaria* metabolites | 56.43±6.26a | 69.59±8.92ab |
| *Purpureocillium lilacinum* metabolites | 64.31±13.96a | 62.07±6.98b |
| Control | 29.55±0.8b | 56.21±4.52b |
| *P*-value | 0.011 | 0.004 |
| *F*-value (df) | 7.42 (3,8) | 10.15 (3,8) |

Values are presented as mean ± SD. Within each column, means followed by different superscript letters are significantly different (ANOVA followed by Tukey’s HSD test, p < 0.05).
